# A pro-apoptotic function of iASPP by stabilizing p300 and CBP through inhibition of BRMS1 E3 ubiquitin ligase activity

**DOI:** 10.1038/cddis.2015.17

**Published:** 2015-02-12

**Authors:** D Kramer, M Schön, M Bayerlová, A Bleckmann, M P Schön, M Zörnig, M Dobbelstein

**Affiliations:** 1 Department of Molecular Oncology, University Medical Center Göttingen, Göttingen, Germany; 2 Department of Dermatology, Venereology and Allergology, University Medical Center Göttingen, Göttingen, Germany; 3 Department of Medical Statistics, University Medical Center Göttingen, Göttingen, Germany; 4 Department of Hematology and Medical Oncology, University Medical Center Göttingen, Göttingen, Germany; 5 Institute of Tumor Biology and Experimental Therapy, Georg Speyer Haus, Frankfurt am Main,Germany

## Abstract

The p53 family and its cofactors are potent inducers of apoptosis and form a barrier to cancer. Here, we investigated the impact of the supposedly inhibitory member of the apoptosis-stimulating protein of p53, iASPP, on the activity of the p53 homolog TAp73, and its cofactors p300 and CBP. We found that iASPP interacted with and stabilized the histone acetyltransferase p300 and its homolog CBP upon cisplatin treatment. Vice versa, iASPP depletion by shRNA resulted in decreased amounts of p300 and CBP, impaired binding of p300 and TAp73 to target site promoters, reduced induction of pro-apoptotic TAp73 target genes, and impaired apoptosis. Mechanistically, we observed that the p300-regulatory E3 ubiquitin ligase BRMS1 could rescue the degradation of p300 and CBP in cisplatin-treated, iASPP-depleted cells. This argues that iASPP stabilizes p300 and CBP by interfering with their BRMS1-mediated ubiquitination, thereby contributing to apoptotic susceptibility. In line, iASPP overexpression partially abolished the interaction of BRMS1 and CBP upon DNA damage. Reduced levels of iASPP mRNA and protein as well as CBP protein were observed in human melanoma compared with normal skin tissue and benign melanocytic nevi. In line with our findings, iASPP overexpression or knockdown of BRMS1 each augmented p300/CBP levels in melanoma cell lines, thereby enhancing apoptosis upon DNA damage. Taken together, destabilization of p300/CBP by downregulation of iASPP expression levels appears to represent a molecular mechanism that contributes to chemoresistance in melanoma cells.

Tumor suppressor proteins of the p53 family, that is, p53, TAp73, and TAp63, mediate cell-cycle arrest and apoptosis through transcriptional regulation of their target genes.^1, 2^ On the other hand, in a majority of solid tumors, p53 function is impaired due to mutations in the TP53 gene or modulations in upstream signaling pathways to p53.^3^ However, in many of these tumors, the p53-family member TAp73 can still mediate cell growth arrest or apoptosis by regulating a set of target genes that overlaps with p53-responsive genes.^4^ Upon treatment of tumors with chemotherapeutics, such as cisplatin or etoposide, TAp73 becomes activated, followed by the transcriptional upregulation of pro-apoptotic p73 target genes and induction of tumor cell apoptosis.^5, 6, 7^ TAp73 functions are tightly regulated by cofactor proteins.^8^ The apoptosis stimulating proteins of p53 (ASPP family) constitute one important group of such cofactors that control p53 family-mediated apoptosis.^9^ In contrast to the two pro-apoptotic members ASPP2 and ASPP1, iASPP was reported to represent an inhibitory member of the ASPP family.^10^

iASPP undergoes multiple protein–protein interactions due to its SH3 domains, ankyrin repeats, and a proline-rich region.^10^ Overexpression of iASPP was previously implied in the inhibition of p53 activity, dependent on the cellular context and stimulus.^11^ Physical interaction of iASPP and TAp73 was observed using recombinant proteins.^12^ Moreover, iASPP depletion was reported to trigger p73-dependent apoptosis in otherwise untreated cells.^13^ However, the impact of iASPP on the activation of TAp73 by chemotherapy remains elusive.

Another important class of cofactors for p53 and TAp73 is represented by the KAT3 family of histone acetyltransferases. Two members of this family, p300 and CBP, acetylate histones as well as non-histone proteins.^14^ Upon DNA damage, p53 and TAp73 become acetylated by p300/CBP, which leads to their stabilization and activation as transcription factors.^15, 16^ As a consequence, p300-p53 family complexes localize to the promoters of pro-apoptotic target genes, such as BBC3/Puma or CD95/Fas, causing their transcriptional upregulation and ultimately trigger apoptosis.^17, 18^ This pro-apoptotic activity of p300/CBP is tightly regulated, and accordingly, the E3 ubiquitin ligase BRMS1 poly-ubiquitinates and thereby destabilizes p300.^19^

iASPP binds not only p53 family members, but also p300.^20^ However, the functional consequences of these interactions are still unclear. Therefore, we assessed the impact of iASPP on the function of p300/CBP as cofactors of TAp73 in DNA-damaged cells.

We observed that iASPP enhanced the stability of p300 and CBP after DNA damage. Consequently, iASPP-depleted cells poorly induced p73-responsive genes and partially failed to undergo apoptosis upon cisplatin treatment. Mechanistically, binding of iASPP to p300/CBP inhibited the functional interaction of p300/CBP with their E3 ubiquitin ligase BRMS1 after DNA damage. Interestingly, iASPP was expressed at low levels in melanoma biopsies, and its enforced overexpression partially reconstituted apoptotic sensitivity in melanoma cell lines. Hence, reduced iASPP levels might contribute to the intrinsic chemoresistance of melanoma.

## Results

### iASPP binds and stabilizes p300/CBP upon DNA damage

**Figure 1:**
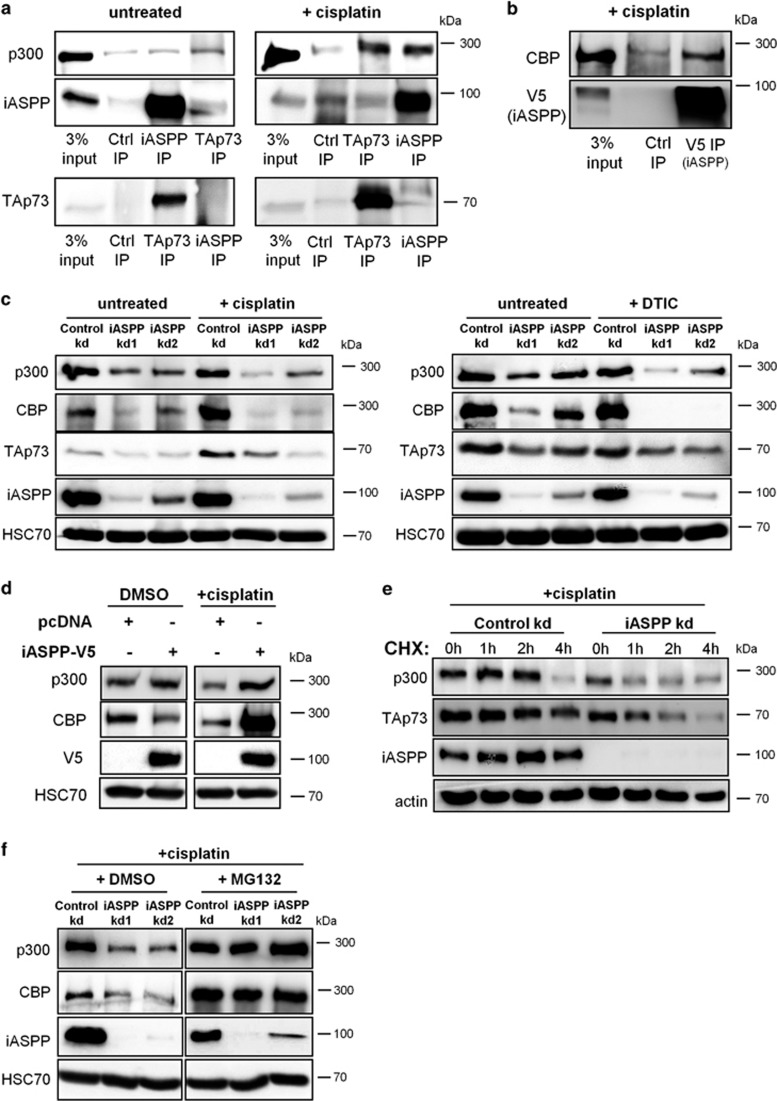
iASPP binds and stabilizes p300/CBP in response to DNA damage. (**a**) iASPP interacts with p300 upon cisplatin treatment. Co-immunoprecipitation of iASPP, p73, and p300 was performed using lysates from untreated or cisplatin-treated HCT116 cells (20 *μ*M cisplatin for 16 h). (Ctrl=control reaction with IgG antibody, IP=Immunoprecipitation). (**b**) iASPP interacts with CBP in cisplatin-treated HCT116 cells. Cells were transiently transfected with an iASPP-V5 expression plasmid or an empty vector control. 24 h after transfection, the cells were treated with 20 *μ*M cisplatin for 24 h, followed by immunoprecipitation of iASPP using an antibody against the V5 tag and immunoblot detection of CBP. (**c**) iASPP knockdown results in decreased p300/CBP protein levels upon cisplatin or dacarbazine treatment. Total protein lysates were prepared from cisplatin-treated (20 *μ*M cisplatin for 24 h) or dacarbazine-treated (1 mM dacarbazine for 24 h) HCT116 cells, followed by immunoblot analysis (DTIC=dacarbazine). (**d**) Overexpression of iASPP in cisplatin- or dacarbazine-treated cells elevates p300/CBP protein levels. The iASPP-V5 expression plasmid or an empty vector control was transiently transfected in HCT116 cells. 24 h after transfection, cells were treated with cisplatin (20 *μ*M) for another 24 h and then processed for immunoblot analysis. (**e**) iASPP decreases the protein stability of p300 and TAp73 upon cisplatin treatment. HCT116 cells were treated for 20 h with 20 *μ*M cisplatin, followed by addition of 100 *μ*g/ml cycloheximide (CHX) for 0 h, 1 h, 2 h, or 4 h. (**f**) Proteasome inhibitor treatment can re-establish p300/CBP levels in iASPP-depleted cells. HCT116 cells containing the indicated shRNA constructs were treated for 20 h with 20 *μ*M cisplatin, followed by 4 h of additional treatment with 10 *μ*M MG132 or DMSO alone

We tested the interactions of endogenous iASPP with p300 and TAp73 in untreated as well as cisplatin-treated HCT116 cells. Under normal growth conditions, we failed to detect any direct interaction of iASPP with p300 or TAp73 by co-immunoprecipitation (Figure 1a). However, we observed iASPP-p300 as well as p300-TAp73 protein complexes in cisplatin-treated cells (Figure 1a), a DNA-damaging condition that can result in activation of p300/CBP through multiple phosphorylation events of both proteins.^21, 22^ Moreover, we detected an interaction of transiently overexpressed iASPP with endogenous CBP, a p300 paralog, upon cisplatin treatment (Figure 1b).

Next, we assessed the impact of iASPP depletion on p300 and CBP expression levels. Since p300 can acetylate and stabilize TAp73 after exposure of cells to cisplatin,^15, 23^ we tested the impact of iASPP on TAp73 expression levels as well. We generated stable iASPP knockdown cells using two different shRNAs against iASPP and quantified the levels of p300, CBP, and TAp73. Upon cisplatin treatment, iASPP-depleted cells displayed reduced amounts of p300, CBP, and TAp73, whereas a detectable but less pronounced impact of iASPP knockdown on the levels of p300/CBP and TAp73 was observed in the absence of chemotherapeutics (Figure 1c). Similar results were obtained when the cells were treated with dacarbazine (DTIC), an alkylating agent (Figure 1c). In contrast, the mRNA levels from CBP and p300 remained unaffected by iASPP depletion (Supplementary Figure S1A). *Vice versa*, transient overexpression of iASPP increased the amounts of p300 and CBP proteins in cisplatin-treated cells (Figure 1d), whereas the corresponding mRNA levels remained the same (Supplementary Figure S1B). Cycloheximide chase experiments in cisplatin-treated HCT116 cells revealed a faster degradation of p300 upon iASPP knockdown compared with control shRNA (Figure 1e, Supplementary Figure S1C). We conclude that iASPP enhances the stability of p300 in cisplatin-treated cells. Similar to the effects of iASPP depletion on p300 stability, iASPP knockdown resulted in a faster degradation of TAp73 in cisplatin-treated cells (Figure 1e, Supplementary Figure S1C). The proteasome inhibitor MG132 restored the levels of p300 and CBP in cisplatin-treated, iASPP-depleted cells (Figure 1f). Hence, iASPP inhibits the proteasomal degradation of p300/CBP in DNA-damaged cells, thereby also resulting in enhanced stability of TAp73.

### iASPP enhances the association of p300 and TAp73 with the promoter regions of p73 target genes

**Figure 2:**
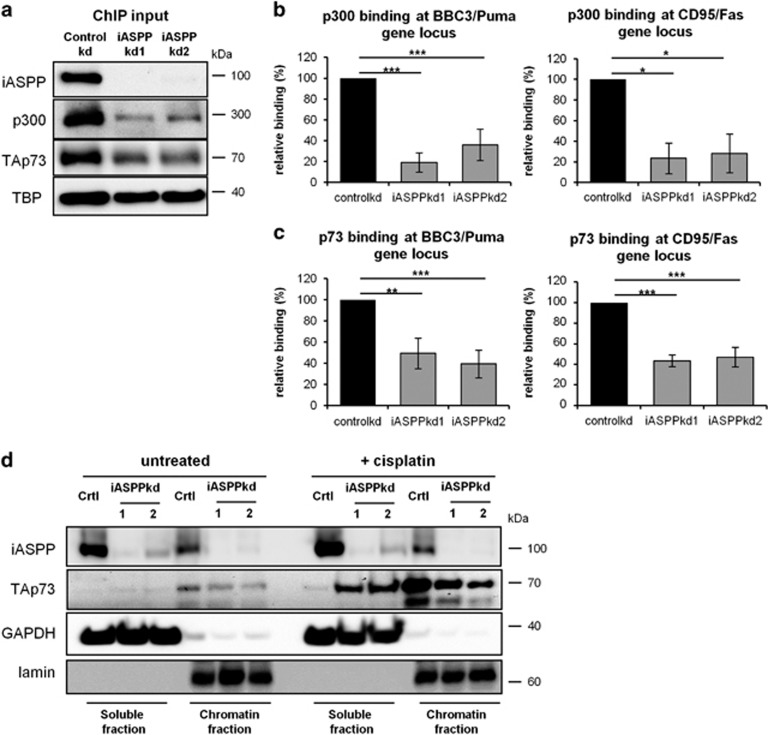
iASPP enhances the association of p300 and TAp73 with the promoter regions of p73 target genes. (**a**) Levels of chromatin-bound p300 and TAp73 decrease in iASPP-depleted cells upon cisplatin treatment. HCT116 cells were treated for 8 h with 20 *μ*M cisplatin before chromatin preparation and immunoblot analysis. (**b**) p300 binds poorly to p300/p73 target sites in iASPP-depleted cells. P300- or IgG-ChIP as control was performed in cisplatin-treated HCT116 cells (8 h, 20 *μ*M cisplatin), followed by quantification of the percentage of precipitated DNA from specific genomic loci. The relative binding describes the fold enrichment of p300 over IgG control. Results from the control knockdown were set to 100% and the relative loss of p300 binding in iASPP knockdown cells was calculated. The average binding from three independent experiments is shown. Significance was determined by the Student's *t*-test and the *P*-value is shown by asterisks (*P-value<0.05, **P-value<0.01, ***P-value<0.001). A detailed ChIP analysis of one biological with all controls is shown in Supplementary File S2. (**c**) iASPP depletion causes a decrease of TAp73-binding to its specific target gene promoters. P73- or IgG ChIP as well as the subsequent analysis was performed as in (**b**). (**d**) iASPP knockdown results in a shift of TAp73 from the chromatin bound to the soluble fraction upon cisplatin treatment. HCT116 cells were treated with cisplatin (16 h, 20 *μ*M cisplatin) before separation of cell lysates into triton-soluble (soluble fraction) and triton-insoluble (chromatin-bound) fractions

Acetylation of histones within chromatin is a major function of p300. We therefore tested the impact of iASPP on the amount of chromatin-associated p300 and TAp73, by chromatin fractionation as well as chromatin immunoprecipitation (ChIP) from cisplatin-treated HCT116 cells. Lower levels of p300 and TAp73 were found in the chromatin fraction of cisplatin-treated, iASPP-depleted cells compared with control cells (Figure 2a). ChIP assays revealed diminished binding of p300 to known p73 target sites, for example, the BBC3/Puma and the CD95/F as promoter (Figure 2b, Supplementary Figure S2A). In parallel, we observed reduced TAp73 binding to its target gene promoters in cisplatin-treated, iASPP-depleted cells as well (Figure 2c, Supplementary Figure S2B). Interestingly, p53 binding to similar promoter regions remained unaffected by iASPP depletion (Supplementary Figure S2C). Moreover, TAp73 protein re-localized from the chromatin bound to the soluble fraction in iASPP knockdown cells (Figure 2d). In conclusion, iASPP is required for the efficient binding of p300 and TAp73 to their target sites in the context of DNA damage.

### iASPP augments TAp73/p300-mediated gene expression

**Figure 3:**
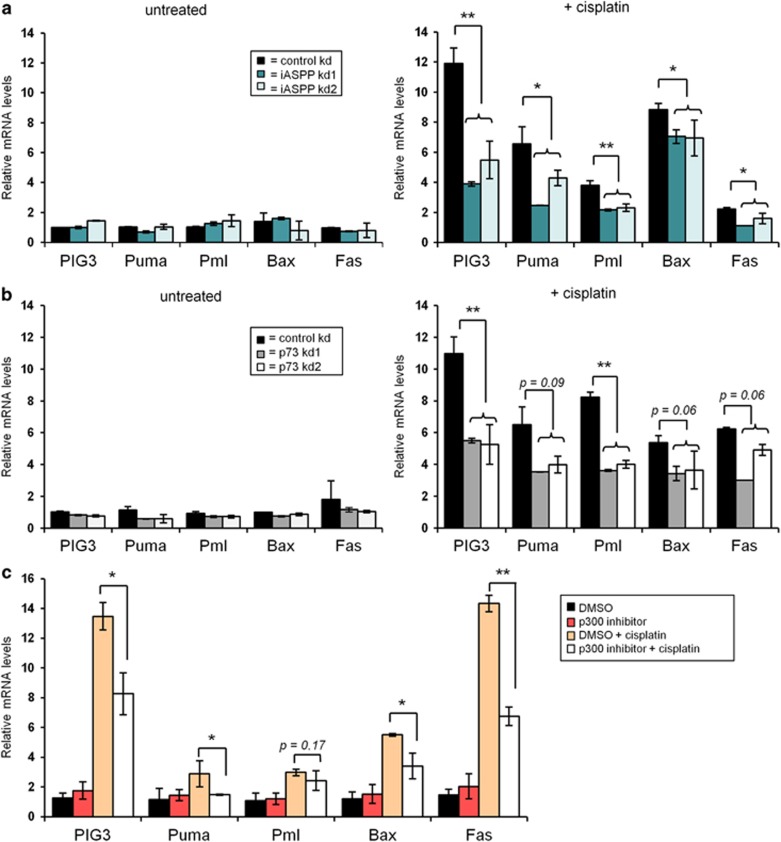
iASPP is required for efficient p73/p300-mediated gene expression upon cisplatin treatment. (**a**) iASPP knockdown results in lower induction levels of pro-apoptotic target genes. HCT116 cells, transduced to synthesize iASPP shRNA or control shRNA, were treated for 12 h with 20 *μ*M cisplatin. Gene expression was analyzed by real-time qPCR. Values are normalized to the reference gene HPRT1. The bar graph represents the relative mRNA levels compared with untreated control knockdown cells. The *P*-value is calculated using the Student's *t*-test and significance is indicated by asterisks as in Figure 2. (**b**) p73 knockdown by shRNA results in a comparable reduction in the mRNA levels of pro-apoptotic target genes after cisplatin treatment. The analysis was performed as in (**a**). (**c**) Inhibition of p300 activity decreases mRNA levels of p73 target genes after cisplatin treatment. HCT116 cells were pre-treated with DMSO or 40 *μ*M of the p300-inhibitor C646 for 24 h followed by treatment with 20 *μ*M cisplatin for 24 h and subsequent cDNA synthesis and real-time qPCR analysis. The bar graph represents the relative mRNA levels normalized to DMSO-treated cells. The *P*-values were calculated using the Student's *t*-test, and the significance is indicated by asterisks as in Figure 2b. The Significance was determined by Student's *t*-test, and the *P*-values are shown by asterisks (*P-value <0.05, **P-value <0.01)

TAp73 induces the expression of pro-apoptotic target genes after cisplatin treatment.^5, 17^ Therefore, we tested the consequences of iASPP knockdown on the expression of these genes and found a significant decrease in their induction levels upon cisplatin treatment (Figure 3a). Knockdown of TAp73 exerted a similar effect on pro-apoptotic p73 target genes (Figure 3b). iASPP knockdown in HCT116−/− p53 cells revealed a comparable decrease in the mRNA levels of the same group of target genes, thereby excluding an impact of p53 expression on the observed effects (Supplementary Figure S3A). In contrast, BH3-only genes (Bim, Bak, Bcl-2) were not downregulated in their expression levels upon TAp73 or iASPP knockdown (Supplementary Figure S3B), further arguing that iASPP specifically regulates TAp73-dependent gene regulation. Finally, upon treatment of HCT116 cells with the p300 inhibitor C646, we observed that the mRNA levels of p73-responsive genes were significantly reduced (Figure 3c), whereas pre-treatment with CTB, a p300 activating drug, partially rescued the expression of these genes upon iASPP knockdown and cisplatin treatment (Supplementary Figure S3C). Taken together, these results indicate that iASPP is indeed involved in the transcriptional upregulation of TAp73/p300 target genes upon cisplatin-mediated DNA damage, independently of p53 expression. Similar results were obtained for etoposide-treated cells (Supplementary Figures S4A and B).

### iASPP contributes to p300-mediated apoptosis

**Figure 4:**
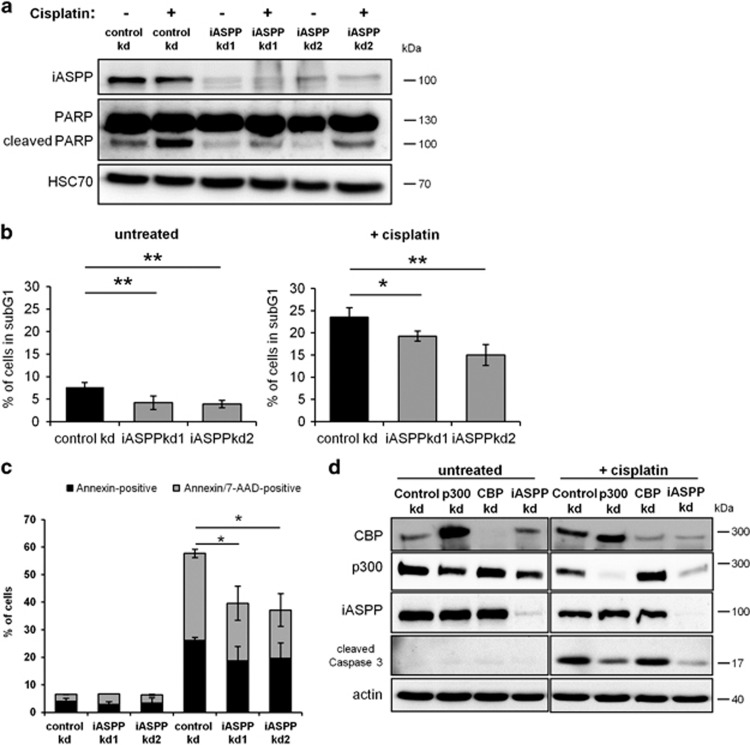
iASPP contributes to p300-mediated apoptosis. (**a**) iASPP knockdown results in lower levels of cleaved PARP in cisplatin-treated cells. HCT116 cells were treated for 24 h with 40 *μ*M cisplatin. Cell lysates were harvested and subjected to immunoblot analysis. (**b**) Depletion of iASPP leads to lower amounts of apoptotic cells after cisplatin treatment. HCT116 cells were left untreated or incubated with 40 *μ*M cisplatin for 24 h before harvest and ethanol fixation. The cells were stained with propidium iodide, and their DNA content was determined by flow cytometry to assess the percentage of cells in the sub-G1 fraction that corresponds to apoptotic cells. Identical gate settings were applied for all samples analyzed. The significance levels were calculated as in Figure 2b. (**c**) iASPP knockdown decreases Annexin V staining upon cisplatin treatment. HCT116 cells were left untreated or incubated with 40 *μ*M cisplatin for 24 h before harvest and staining of the cells with Annexin V/ 7-AAD solution. The percentages of unstained, Annexin V-positive, and Annexin V/7-AAD-positive cells were determined using flow cytometry. Identical gate settings were applied for all samples. The mean and the standard error derived from three biological replica. *P*-values were calculated as in Figures 2b. (**d**) Cisplatin-mediated apoptosis partially depends on p300. In HCT116 cells containing control shRNA, p300 or CBP was transiently knocked down using siRNA. For control, scrambled siRNA was applied. 48 h after transduction, the cells were treated with 40 *μ*M cisplatin for 24 h. In parallel, cells with a stable iASPP shRNA expression construct were treated and analyzed. Note that the depletion of CBP led to a compensatory increase in p300 levels, perhaps explaining why CBP knockdown did not grossly affect the extent of caspase cleavage. The Significance was determined by Student's *t*-test, and the *P*-values are shown by asterisks (*P-value <0.05, **P-value <0.01)

Since pro-apoptotic TAp73 target gene expression depends on iASPP, we tested the impact of iASPP depletion on the induction of cisplatin-mediated apoptosis. Depletion of iASPP and subsequent cisplatin treatment of HCT116 cells induced lower levels of cleaved PARP compared with control knockdown cells, indicating a reduced level of apoptosis (Figure 4a). In parallel, propidium iodide staining and flow cytometry of cisplatin-treated HCT116 cells revealed a significantly lower percentage of the apoptotic sub-G1 population upon iASPP knockdown, compared with control shRNA (Figure 4b). Along the same line, the percentage of Annexin V-positive HCT116 cells with or without p53 was reduced upon iASPP knockdown and cisplatin treatment (Figure 4c, Supplementary Figure S5A). Similar results were obtained for etoposide-treated, iASPP-depleted HCT116 cells (Supplementary Figure S5B). These results indicate that iASPP depletion results in a decrease of cisplatin-mediated apoptosis, independently of p53. Moreover, transient knockdown of p300 in HCT116 cells resulted in a similar reduction of cleaved Caspase-3 compared with iASPP knockdown (Figure 4d). In conclusion, iASPP contributes to cisplatin-induced, p300-driven apoptosis, in agreement with its role in the induction of pro-apoptotic genes.

### BRMS1 destabilizes p300 and CBP in the absence of iASPP

**Figure 5:**
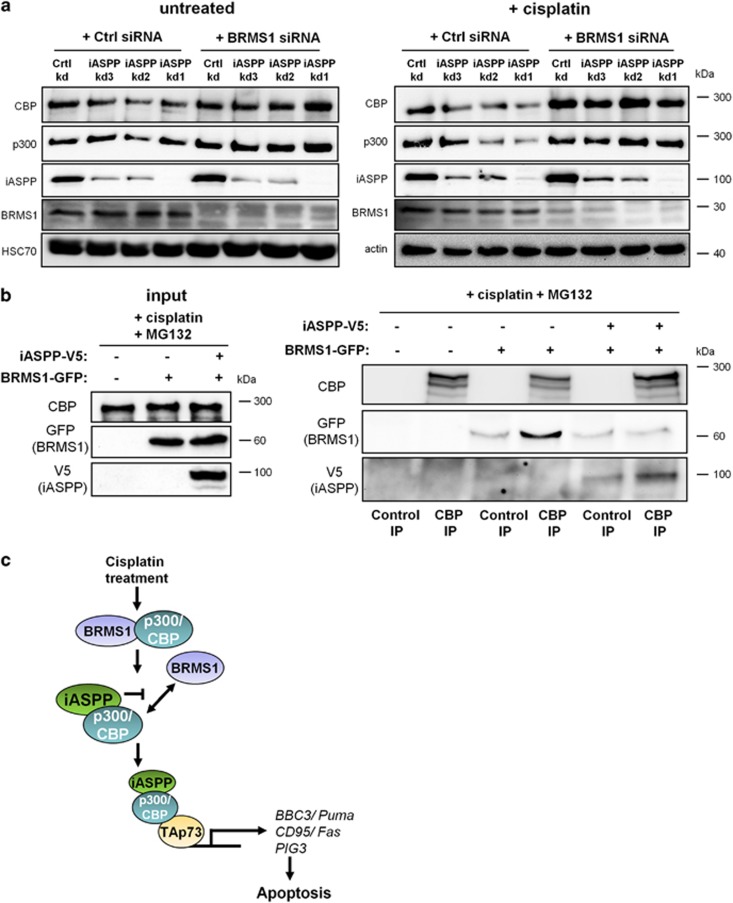
BRMS1 mediates the destabilization of p300 and CBP upon iASPP removal and cisplatin treatment. (**a**) BRMS1 knockdown can rescue p300 and CBP levels in cisplatin-treated, iASPP-depleted cells. BRMS1 was transiently knocked down with siRNA in cells that expressed control or iASPP shRNA. Scrambled siRNA was transfected in parallel for control. 48 h after knockdown, the cells were either left untreated or incubated with cisplatin (24 h, 20 *μ*M) followed by immunoblot analysis. (**b**) iASPP overexpression abrogates the interaction of BRMS1 and CBP in cisplatin-treated cells. HCT116 cells were transiently transfected with a BRMS1-GFP expression construct, alone or in combination with the iASPP-V5 expression plasmid. 24 h after transfection, the cells were treated with 20 *μ*M cisplatin for 16 h and 20 *μ*M MG132 for the last 4 h. Co-immunoprecipitation was performed using either an antibody for CBP or an isotype-matched control antibody (IgG). (**c**) Proposed mechanism of iASPP-mediated apoptosis. iASPP inhibits BRMS1-dependent proteasomal degradation of p300/CBP, thereby enhancing the induction of pro-apoptotic TAp73 target genes

Given that breast metastasis suppressor 1 (BRMS1) was described as an E3 ubiquitin ligase for p300,^19^ we tested whether BRMS1 was responsible for p300/CBP destabilization in iASPP-depleted cells. Indeed, upon cisplatin treatment, a transient knockdown of BRMS1 restored the diminished protein levels of p300 and CBP in iASPP-depleted cells (Figure 5a). This suggested an inhibitory role for iASPP in the BRMS1-mediated destabilization of p300 and CBP.

We then analyzed whether overexpressed iASPP interfered with the interaction between BRMS1 and p300/CBP in cisplatin-treated HCT116 cells. Indeed co-immunoprecipitation of CBP and BRMS1, in the absence *versus* presence of an iASPP expression plasmid, showed that an excess of iASPP reduced the interaction of CBP and BRMS1 (Figure 5b). Taken together, our results suggest a model (Figure 5c) according to which iASPP reduces the access of BRMS1 to p300/CBP. iASPP thereby enhances p300/CBP levels and activity, and ultimately promotes TAp73-induced apoptosis.

### Melanomas are characterized by downregulated iASPP and CBP expression

**Figure 6:**
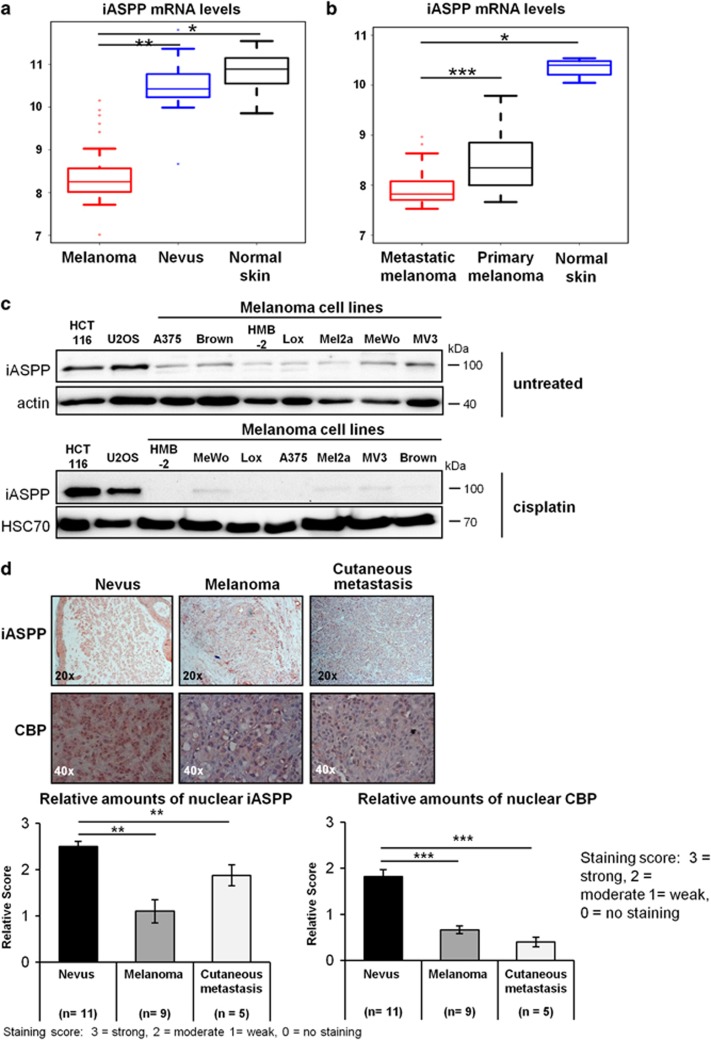
Malignant melanoma is characterized by downregulated iASPP and CBP expression. (**a**) iASPP expression is strongly downregulated in primary melanoma compared with benign nevi and normal skin tissue. The gene expression study was applied on 45 primary melanomas, 18 melanocytic nevi, and 7 normal skin tissue sections.^24^ The published microarray data (GSE3189) were re-analyzed for gene expression of the PPP1R13L gene that encodes iASPP. Significance was calculated with a two-sided Student's *t*-test and is represented by asterisks, similar to Figure 2. (**b**) iASPP expression is significantly downregulated in metastatic melanomas compared with normal skin tissue. The original study was published by Riker *et al.*,^25^ using 40 metastatic melanomas, 42 primary cutaneous melanomas and 4 normal skin tissue sections. The microarray data (GSE7553) were re-analyzed for relative mRNA levels of iASPP encoded by the PPP1R13L gene as in (**a**). (**c**) Untreated and cisplatin-treated melanoma cell lines display low levels of iASPP. Seven different melanoma cell lines (A375, Brown, Lox, HMB-2, Mel2a, MeWo, and MV3 cells) and two cell lines from different cancer species, HCT116 (colon adenocarcinoma cells) and U2OS (osteosarcoma cells) were analyzed by immunoblot for iASPP protein levels, untreated or after cisplatin treatment (24 h, 20 *μ*M cisplatin). A comparison of iASPP protein levels between melanoma-derived cells and primary melanocytes is shown in Supplementary Figure S6A. (**d**) Human melanomas display reduced protein levels of nuclear iASPP and CBP *in situ*. Complete excision biopsies of 11 benign nevi (compound nevi with both epidermal and prominent dermal portion), 9 primary melanomas, and 5 cutaneous melanoma metastases were subjected to immunohistochemical staining of iASPP and CBP. All samples were stained in parallel and evaluated for their relative staining intensity in the cytoplasm *versus* the nucleus. The staining intensities were evaluated using a semi-quantitative score ranging from 0 for no staining to 3 for high staining intensity. The evaluation was carried out in a 'blinded' manner by a dermatopathologist unaware of the identity of the samples. The bar graphs show the relative nuclear staining intensities of iASPP and CBP. Significance was calculated using the Student's *t*-test and is illustrated by asterisks (*P-value<0.05, **P-value<0.01, ***P-value<0.001)

The notion that iASPP can act as a pro-apoptotic cofactor of p300/p73-mediated apoptosis, raised the hypothesis that some tumor entities might benefit from low iASPP expression levels and the resulting impairment of p300/CBP function. And indeed, iASPP mRNA levels were reduced about 4-fold in a panel of 45 malignant melanoma samples, compared with benign nevi and normal skin tissue according to GEO data set GSE3189 (Figure 6a).^24^ Comparison of primary melanomas, samples of metastatic melanomas and normal skin tissue of another study (GSE7553) confirmed that iASPP expression was reduced to a similar degree in primary melanoma and further decreased in metastatic melanoma (Figure 6b).^25^ Accordingly, the analysis of seven untreated or cisplatin-treated melanoma-derived cell lines revealed that iASPP is present at lower levels in melanoma cell lines compared with cell lines from different tumor species (Figure 6c). Moreover, melanoma-derived cell lines revealed decreased levels of p300 and iASPP when compared with normal human epidermal melanocytes (NHEMs) (Supplementary Figure S6A). In line with our proposed model of iASPP-mediated inhibition of p300/CBP degradation, immunohistochemical analysis revealed a significant decline in the nuclear protein levels of CBP and iASPP in 9 primary melanomas and 5 cutaneous melanoma metastases, compared with 11 benign melanocytic nevi (Figure 6d, Supplementary Figures S6B, C, S7A and B).

### iASPP and BRMS1 modulate CBP/p300 levels in melanoma

**Figure 7:**
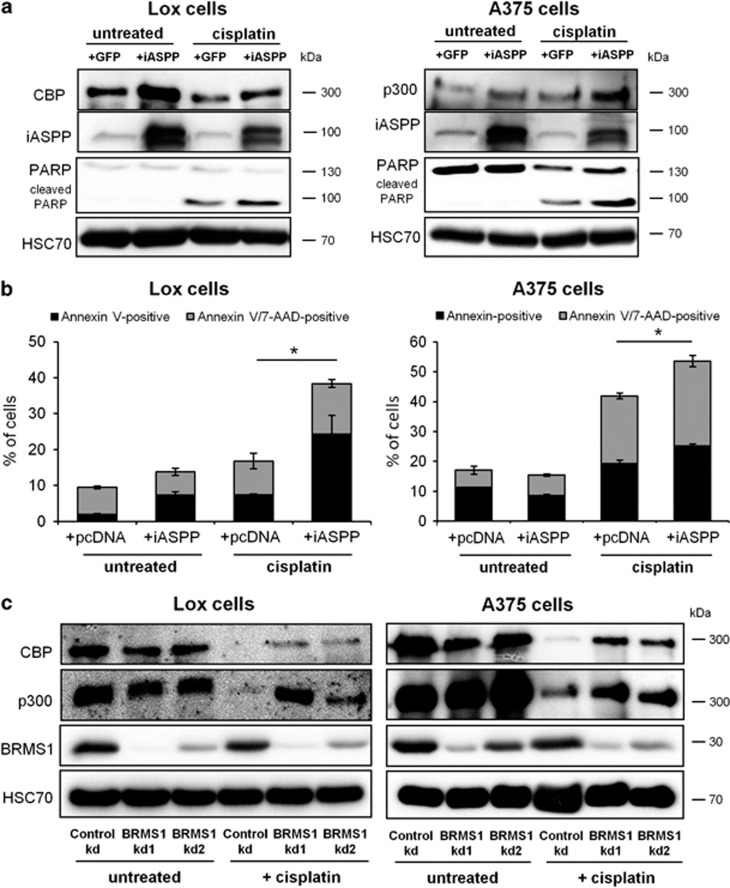
iASPP and BRMS1 modulate p300/CBP levels and apoptosis in melanoma. (**a**) Overexpression of iASPP partially re-establishes p300/CBP levels in melanoma cells. The iASPP-V5 expression plasmid or a GFP control vector was transiently transfected into Lox and A375 cells. 24 h after transfection, the cells were treated with 10 *μ*M cisplatin for 24 h. Subsequently, total protein lysates were prepared and analyzed by immunoblotting. (**b**) Overexpression of iASPP enhances cisplatin-mediated apoptosis in melanoma cells. An expression plasmid for iASPP-V5 or the control vector pcDNA3 was transiently transfected into Lox and A375 cells. 24 h after transfection, the cells were treated with 20 *μ*M cisplatin for 24 h, followed by harvest of the cells and staining with Annexin V/7-AAD solution. The percentages of unstained, Annexin V-positive and Annexin V/7-AAD-positive cells were determined using flow cytometry as in Figure 4c. Significance was calculated as in Figure 6d. (**c**) BRMS1 knockdown elevates p300 and CBP protein levels in Lox and A375 cells. BRMS1 was knocked down using siRNA. Scrambled siRNA was transfected in parallel as a control. 48 h after transfection, the cells were treated with 10 *μ*M cisplatin, and immunoblots were stained for p300 and CBP. The Significance was determined by Student's *t*-test, and the *P*-values are shown by asterisks (*P-value <0.05)

According to the model proposed in Figure 5c, downregulation of iASPP in melanoma cells would confer a reduction in CBP/p300 expression levels, as well as increased chemoresistance upon cisplatin treatment. To test this hypothesis, we transiently overexpressed iASPP in melanoma cell lines, followed by cisplatin treatment. Indeed, restoration of iASPP levels in melanoma cells consistently elevated CBP/p300 protein levels in cisplatin-treated Lox and A375 cells (Figure 7a). In Lox cells, where endogenous p73 was detectable, its levels were also upregulated upon re-expression of iASPP (Supplementary Figure S8). Moreover, iASPP overexpression increased the extent of apoptosis in cisplatin-treated cells, as revealed by the detection of PARP cleavage and Annexin V/7-AAD staining (Figures 7a and b). Similarly, transient depletion of BRMS1 partially re-established p300/CBP levels in cisplatin-treated melanoma cell lines (Figure 7c). We conclude that the reduction of iASPP expression in melanoma facilitates BRMS1-mediated p300/CBP turnover, thereby contributing to chemoresistance.

## Discussion

iASPP was hitherto regarded as an inhibitor of apoptosis, antagonizing the pro-apoptotic activities of p53 and its cousin TAp73.^11, 13^ In contrast to this view, our results uniformly point to an unexpected pro-apoptotic role for iASPP upon DNA damage. This function is carried out at least in part by the interaction of iASPP with the acetyltransferases p300 and CBP, displacing the antagonizing ubiquitin ligase BRMS1. As a consequence, iASPP promotes the stability of p300/CBP and supports their function as cofactors of TAp73. Thereby, TAp73-mediated transactivation of pro-apoptotic genes is enhanced.

This model (summarized in Figure 5c) conspicuously contrasts with previous reports describing iASPP as an antagonist of other ASPP family proteins and their pro-apoptotic activities.^11, 26^ The discrepancy might be explained by the different experimental settings and cell systems that were used in previous studies. Moreover, we propose that the differential activities of p300/CBP upon a variety of stimuli form the basis for the pro- or anti-apoptotic activities of iASPP. For instance, p300 and CBP represent essential cofactors not only for pro-apoptotic p53 family members, but also for NF-*κ*B, a group of transcription factors that are mostly known for their pro-survival activities.^27, 28^ With this perspective, it seems less of a surprise that enhancing p300 and CBP activities by iASPP can affect cell survival in opposite ways, dependent on the cell type and specific stress conditions. Moreover, iASPP was identified as a binding partner of NF-κB family members,^29, 30^ thereby further strengthening the point that it not only regulates pro-apoptotic transcription. Instead, the balance between activities of the p53 and the NF-κB family members may represent a central determinant of how ASPP proteins, as well as p300/CBP, affect cell fate.

Most of the previous studies observed iASPP-mediated anti-apoptotic effects; however, these effects were noted in the absence of DNA-damaging drugs and/or upon iASPP overexpression.^11, 13^ In contrast, we found inhibition of apoptosis after depletion of endogenous iASPP in DNA-damaged tumor cells. DNA damage activates TAp73, and this context appears to involve iASPP as a cofactor rather than an inhibitor. In support of our findings, a recent publication reported that the loss of endogenous iASPP expression in UV-irradiated keratinocytes resulted in the inhibition of apoptosis as well.^31^ However, this report did not address the roles of BRMS1, p300/CBP, or TAp73 in this context. Of note, another key member of the ASPP family, ASPP1, has recently been described as an inhibitor of apoptosis,^32^ in contrast with its previously reported role as a promoter of cell death.^9^ This further illustrates that the function of ASPP family members in cell death or survival is complex and can vary depending on the biological system.

Taken together, our results suggest that iASPP has greater functional similarities to its cousins ASPP1 and ASPP2 than previously anticipated. This finding is also supported by the fact that all three ASPP family members have a similar structural composition. All three contain a proline-rich region, ankyrin repeats, and SH3 domains. These domains are responsible for most if not all known interactions between ASPP and other regulatory proteins.^10^ The only structural feature not shared by iASPP is the presence of an *α*-helical, N-terminal domain with unknown function.^33^ All three ASPP-family members can physically interact with p300, although specific interaction sites between ASPP and p300 have not been elucidated yet.^20^ Thus, if iASPP can sterically hinder the interaction of BRMS1 and p300/CBP, leading to the accumulation of the latter, it is conceivable that all three ASPP family members might be able to perform this action.

We observed the strongest interactions of iASPP with p300 and CBP upon cisplatin treatment. Why does DNA damage enhance this interaction? Although the reasons remain to be elucidated, we hypothesize that the DNA damage response leads to post-translational modifications on either or both of the proteins, thus enhancing their interaction. Indeed, DNA damage induces MAPK- and AKT-mediated phosphorylation of p300 and enhances p300 activity, leading to apoptosis induction.^21, 22^ Therefore, it is possible that one or several DNA-damage-mediated post-translational modifications of p300/CBP are needed for iASPP-p300/CBP complex formation.

The pro-apoptotic function of iASPP is in accordance with its reduced synthesis in melanoma cells. In contrast, a recent publication described iASPP as an inhibitor of p53 and a mediator of increased chemoresistance in metastatic melanoma.^34^ However, these findings do not necessarily contradict our results, since they describe a specific subset of melanoma with sustained iASPP expression and concomitant activation of cyclin B1 and CDK1. CDK1 activity then leads to iASPP monomerization. It is conceivable that in this special situation, iASPP adopts anti-apoptotic functions and loses its co-activator function for p300/CBP. Accordingly, we observed a significant decrease in the nuclear protein levels of iASPP and CBP when comparing primary melanomas and cutaneous melanoma metastases with benign melanocytic nevi (cf. Figure 6). Interestingly, the protein levels of p300 inversely correlate with tumor progression in melanoma.^35^ This argues that p300 and/or CBP may contribute to the suppression of melanoma growth with particularly high efficiency, making it necessary for the tumor cells to suppress the synthesis of a p300/CBP agonist. In support of this hypothesis, losses of heterozygosity of the genes encoding for p300 and CBP are frequently detected in melanoma cell lines.^36^

Our results, arguing that iASPP acts in a pro-apoptotic manner at least in a subset of tumors, should give rise to caution toward the previously proposed strategy of using iASPP as an anti-cancer drug target.^11, 37^ On the other hand, it may be promising to mimic the pro-apoptotic actions of iASPP in situations where it is silenced, especially in melanomas. Like in the case of p14ARF, an inhibitor of the E3 ubiquitin ligase Mdm2, pharmacological antagonists of gene silencers, for example, histone deacetylases (HDACs) or DNA methyltransferases, may act in part through augmenting iASPP levels and thereby preventing BRMS1-mediated p300/CBP-degradation. A similar net effect may be achievable by proteasome inhibition, directly enhancing the amounts of p300/CBP. More specific upregulation of p300/CBP could be expected from targeting BRMS1 directly, but this would first require the development of such pharmacologically active compounds.

Even if the BRMS1-iASPP-p300/CBP-TAp73 axis proved to be difficult to target, it may nonetheless provide prognostic or predictive markers for cancer progression. However, the most upstream component of this regulatory pathway, BRMS1, apparently has dual roles that make its activities difficult to interpret. BRMS1 is known as a suppressor of metastasis (but not of primary tumor progression), presumably through its interaction with the mSIN3a repressor complex.^38^ Accordingly, high cytoplasmic BRMS1 levels represent a favorable prognostic marker regarding the clinical course of melanoma patients,^39^ whereas nuclear BRMS1 staining correlates with a reduced overall survival rate.^40^ We propose that BRMS1 still antagonizes p300/CBP-mediated apoptosis in this tumor; however, this function may be carried out even by small amounts of BRMS1, since its antagonist iASPP is largely absent. In other tumors, BRMS1 may need higher expression levels to antagonize p300/CBP-induced apoptosis. And indeed, in breast cancer, high BRMS1 levels were reported as a marker of poor prognosis.^41^ In contrast to BRMS1 and p300, the direct analysis of iASPP levels remains to be evaluated regarding its relevance for prognosis and prediction of therapeutic responses, in melanoma and other human malignancies.

## Materials and methods

### Cell culture and treatment

All cell lines were maintained in Dulbecco's modified Eagle's medium (DMEM) supplemented with 10% fetal bovine serum (FBS) and antibiotics at 37 °C in a humidified atmosphere with 5% CO_2_. For treatment of the cells, cisplatin (1 mg/ml, Teva, Ulm, Germany), dacarbazine (DTIC, 5 mM in DMSO, Sigma-Aldrich, Taufkirchen, Germany), MG132 (10 mM in DMSO, Merck-Calbiochem, Darmstadt, Germany), cycloheximide (CHX, 100 *μ*g/ml in ethanol, Sigma-Aldrich), C646 (100 mM in DMSO, Sigma-Aldrich) or CTB (100 mM in DMSO) was diluted in pre-warmed medium and added to the cells for the indicated periods of time.

### Stable transduction with shRNA expression vectors, siRNA-mediated knockdown and plasmid transfection

For stable transduction of cell lines, HEK293T cells were co-transfected with lentiviral packaging vectors (*pMD2.G* from Addgene (Cambridge, UK) and *pCMV-R8.91* from Plasmidfactory (Bielefeld, Germany)) and *PLKO-shiASPP* plasmids (PLKO.1-TRCN0000022209, TRCN0000022210, and TRCN0000022212), *PLKO-shp73* plasmids (PLKO.1-TRCN0000006507 and TRCN0000006508) or a *PLKO.1-luc* plasmid (SHC007) for control knockdown (all purchased from Sigma-Aldrich). After lentivirus production, HCT116 cells were transduced in the presence of 8 *μ*g/ml polybrene. For stable selection of iASPP-depleted cells, 0.5 *μ*g/ml puromycin was added to the medium for selection. For transient knockdown of BRMS1 (S24632 and S24633), p73 (S14319), p300 (S4696), or CBP (S3496), 1.5 × 10^5^ cells were transfected with 50 pmol siRNA (silencer select from Life Technologies, Darmstadt, Germany) and Lipofectamine 2000 (Life Technologies). Scrambled siRNA (Cat.4390843, Invitrogen, Darmstadt, Germany) served as a negative control. For transient overexpression of iASPP and BRMS1, 4 × 10^5^ cells were transfected using 4 *μ*l jetPrime transfection reagent (Polyplus, VWR International, Darmstadt, Germany) and 2 *μ*g iASPP-V5 plasmid (gift from Xin Lu, Oxford, UK), 2 *μ*g BRMS1-GFP plasmid (Origene, BioCat GmbH, Heidelberg, Germany), 2 *μ*g GFP expression construct (Origene) or 2 *μ*g pcDNA3.1 control plasmid per 6-well.

### RNA extraction, cDNA synthesis, and gene expression studies

For extraction of total RNA, cells were lyzed using TRIZOL (Life Technologies). After purification, contaminating genomic DNA was digested using DNase I (30 min, 37 °C, Thermo Scientific Fisher, Dreieich, Germany). For cDNA synthesis, 1 *μ*g of RNA was reverse transcribed using random hexamer primers and Revert Aid H^-^ reverse transcriptase (Thermo Scientific Fisher). Real-time qPCR was performed for 40 cycles and with 58 °C annealing temperature using the CFX96 thermocycler (Bio-Rad, München, Germany). Primer sequences are listed in Supplementary Table S1. For calculation of the relative mRNA levels, values of the target genes were normalized to HPRT1 or Actin. The mean log ratio was calculated from the ΔΔCt (−2^−ΔΔCt^). *P*-values were calculated using the Student's *t*-test.

### Protein harvest, immunoblot analysis, and co-immunoprecipitation

Cells were harvested in protein lysis buffer (20 mM TRIS-HCl pH 7.5, 150 mM NaCl, 1 mM Na_2_EDTA, 1 mM EGTA, 1 mM beta-glycerophosphate) with 1/4 volume of 8 M Urea and 1x PIC (Protease inhibitor Cocktail, Roche, Mannheim, Germany) and sonicated to disrupt DNA–protein complexes. Total protein concentration was measured using a Pierce BCA Protein assay kit (Thermo Scientific Fisher). After boiling, the samples with Laemmli buffer at 95 °C for 5 min, equal amounts of protein samples were separated by SDS-PAGE. After separation and transfer of the proteins onto nitrocellulose, proteins were visualized using the following antibodies: iASPP, A4605 (Sigma-Aldrich), p73, ab14430 (Abcam, Cambridge, UK), p300, sc-584 (Santa Cruz, Heidelberg, Germany), CBP, sc-369 (Santa Cruz), TBP, SL30 (Diagenode, Liege, Belgium), HSC70, B-6 (Santa Cruz), *β*-Actin, ab8227 (Abcam), BRMS1, 2D4-2G11 (Abnova, Heidelberg, Germany), ab134968 (Abcam), PARP1, 9542 (Cell Signaling Technology, Frankfurt, Germany), cleaved Caspase-3, 9664 (Cell Signaling Technology), V5, V8012 (Sigma-Aldrich), GAPDH, ab8245 (Abcam), Lamin B, ab16048 (Abcam).

Co-immunoprecipitation was performed as described before.^20^ For immunoprecipitation reaction, 3 *μ*g anti-CBP antibody (sc-369, Santa Cruz) or 3 *μ*g pre-immune IgG antibody (ab46540, Abcam), together with pre-blocked Sepharose G beads, was incubated with the cell lysates, overnight at 4 °C and then processed as above.

### Chromatin immunoprecipitation and chromatin fractionation

Chromatin fractionation was performed as described before.^42^ For subsequent immunoblot analysis, Lamin B1 is a nuclear protein and was detected to control the identity and purity of the chromatin fraction, whereas GAPDH was stained to identify the soluble fraction.

ChIP experiments were conducted as described before.^43^ For each ChIP reaction, chromatin from approximately 2 × 10^6^ cells was incubated with 30 *μ*l protein A/G plus agarose beads (sc-2003) and 2 *μ*g anti-p73 antibody (ab14430, Abcam), 5 *μ*g anti-p300 antibody (sc-584X, Santa Cruz) or corresponding amounts of anti-IgG antibody (ab46540, Abcam). After washing and purification, the ChIP samples were analyzed by real-time qPCR using primers with the sequences listed in Supplementary Table S1. The precipitated amount of DNA is presented as the percentage of input. The promoter region of Myoglobin (MB) served as an internal negative control to exclude unspecific antibody binding to DNA.

### Cell-cycle analysis

For cell-cycle analysis, cells were harvested, fixed in ethanol and finally stained with propidium iodide. Cell-cycle profiles were obtained by flow cytometry using the Guava system (Millipore, Darmstadt, Germany). For the quantification of apoptotic cells as the subG1 shoulder, the same gate settings were applied for all samples. Asterisks represent the significance that was calculated using the Student's *t*-test.

### Quantitative assessment of apoptosis

Annexin V/7-AAD staining was conducted on living cells using the Nexin reagent (Millipore). The percentage of apoptotic cells was determined by flow cytometry using the Guava system (Millipore). For quantification of Annexin V-positive, or Annexin V/7-AAD-positive cells, the same gate settings were applied for all samples. For each experiment, at least three biological replica were analyzed. Asterisks represent the significance that was calculated using Student's *t*-test.

### Histochemistry and immunohistochemistry

Paraffin-embedded complete excision material (stored at the biobank of the Department of Dermatology, University Medical Center Göttingen) from 11 melanocytic compound nevi (i.e., containing both an epidermal and a dermal portion), 9 primary melanomas (Breslow level/vertical tumor thickness >1 mm), and 5 cutaneous melanoma metastases was subjected to histochemistry and immunohistochemistry after obtaining approval by the local ethics committee.

Hematoxylin and eosin staining was performed according to standard protocols. For immunohistochemistry, sections of 3 *μ*m thickness were de-paraffinized and re-hydrated. Thereafter, sections were boiled in *DAKO-target-retrieval-solution* (DAKO, Hamburg, Germany) for 20 min and washed three times with PBS. Endogenous peroxidase was inactivated with 3% H_2_O_2_ for 10 min. Unspecific binding sites were blocked by 5% goat serum (Roth) in PBS containing 0.01% Triton X-100 for 45 min. The tissue samples were incubated overnight at 4 °C with the polyclonal anti-CBP antibody (clone A-22, 1:20, Santa Cruz) or the monoclonal anti-iASPP antibody (clone LXO49.3, Sigma-Aldrich), followed by 60 min incubation with the horseradish peroxidase-labeled secondary anti-rabbit IgG (Promega, Madison, WI, USA). Then, the samples were incubated with 0.2% streptavidin-peroxidase followed by washing steps and 5 min incubation with AEC+-Solution (DAKO), followed by the final haematoxylin (DAKO) staining.

All samples were analyzed and scored for their CBP expression by a dermatopathologist who was 'blinded' regarding the identity of the samples, using an Axioskop200 microscope and the Axiovision software (Zeiss, Oberkochen, Germany). Two primary melanomas had to be excluded from this analysis because their high melanin content did not allow reliable evaluation of specific staining (so-called 'animal type' melanomas). All samples were evaluated at identical magnifications, and images were taken using standardized exposure times.

### Analysis of the microarray datasets

Two Affymetrix data sets were retrieved from the NCBI Gene Expression Omnibus (GEO) data repository.^44^ The first data set (GSE3189) comprises 7 normal skin, 18 nevi, and 45 melanoma samples. From the second data set (GSE7553), we randomly selected 4 normal skin, 14 primary melanoma, and 40 metastatic melanoma samples. Both data sets were pre-processed and quantile normalization was performed. To assess a significance of gene expression change between the sample groups, a two-sided Student's *t*-test was applied. All analyses were performed using the statistical software R (version 2.15.2).^45^

## Abbreviations

DTIC: dacarbazine
CHX: cycloheximide
iASPP: inhibitory apoptosis stimulating protein of p53
BRMS1: breast cancer metastasis gene 1
DMSO: dimethylsulfoxide
CBP: CREB-binding protein
SH3 domain: *scr* homology 3 domain
