# Supplementary material for: A pro-apoptotic function of iASPP by stabilizing p300 and CBP through inhibition of BRMS1 E3 ubiquitin ligase activity
Source: Cell Death Dis. 2015 Feb 12;6(2):e1634–. doi: 10.1038/cddis.2015.17 (PMC4669821; doi:10.1038/cddis.2015.17)
Supplement: Supplementary Table 1 [file cddis201517x1.doc]

**Supplementary Table S1**

List of primer sequences for ChIP and gene expression analysis

| Fas_forward | AGGAATGCACACTCACCAGC | Fas (CD95) |
| --- | --- | --- |
| Fas_reverse | TGGAGATTCATGAGAACCTTGG | Fas (CD95) |
| HPRT1_forward | ATGCTGAGGATTTGGAAAGG | HPRT1 |
| HPRT1_reverse | TCATCACATCTCGAGCAAGAC | HPRT1 |
| Noxa_forward | GCCGCGGGTCGGGAGCGTGT | Noxa (PMAIP1) |
| Noxa_reverse | GCCCCTGTCCCCGCCCCTGT | Noxa (PMAIP1) |
| P21_forward | CTTTCTGGCCGTCAGGAACA | P21 (CDKN1A) |
| P21_reverse | CTTCTATGCCAGAGCTCAACATGT | P21 (CDKN1A) |
| P300_forward | GCGGCCTAAACTCTCATCTC | P300 (EP300) |
| P300_reverse | TGGTAAGTCGTGCTCCAAGTC | P300 (EP300) |
| PIG3_forward | ATGTTAGCCGTGCACTTTGACA | PIG3 (TP53I3) |
| PIG3_reverse | CACGTAGAGGTTTTCCGGTCC | PIG3 (TP53I3) |
| Pml_forward | CCGCAAGACCAACAACAT | PML |
| Pml_reverse | ACTGTGGCTGCTGTCAAG | PML |
| PUMA_forward | GAAACGGAATGGAAAGCTATGAGA | Puma (bbc3) |
| PUMA_reverse | GCAGACCCCATGCCAAATT | Puma (bbc3) |
| CBP_forward | CACATGACGCATTGTCAGGC | CBP (CREBP) |
| CBP_reverse | ATTCCACTAGCTGGAGACCC | CBP (CREBP) |
| Actin_forward | CGACAGGCTGCAGAAGGAG | Actin |
| Actin_reverse | GTACTTGCGCTCAAGAGGAG | Actin |
| Bim_forward | AGCCCAGCACCCATGAGTTGTGAC | Bim |
| Bim_reverse | CTCTGGGCGCATATCTGCAGG | Bim |
| Bak_forward | TGCAACATGGTCTGGAACTC | Bak1 |
| Bak_reverse | TGGTCACCTTACCTCTGCAAC | Bak1 |
| Bcl-2_forward | GCGGGAGTACAAGATGATT | BCL-2 |
| Bcl-2_reverse | GCTTGTAGGCTGAAACAGCA | BCL-2 |
| ChIPpuma_forward | CTTGCTAACTGGCCCACTG | BBC3 |
| ChiIPpuma_reverse | CGGAATGGAAAGCTATGAGAC | BBC3 |
| ChIPFas_forward | AGGGCTTGTCCAGGAGTTC | CD95 |
| ChIPFas_reverse | ACAGGAATTGAAGCGGAAGT | CD95 |
| ChIPmb_forward | CTCATGATGCCCCTTCTTCT | MB |
| ChIPmb_reverse | GAAGGCGTCTGAGGACTTAAA | MB |
| ChIPp21_forward | GCAGATGTGGCATGTGTCC | CDKN1A |
| ChIPp21_reverse | AGTGACTGCACGACCTTGG | CDKN1A |
| ChIPMdm2_forward | GCTGGTCAAGTTCAGACACG | MDM2 |
| ChIPMdm2_reverse | ACAGGTCTACCCTCCAATCG | MDM2 |
| ChIPBax_forward | AGCACTGAGGAGAAAC | BAX |
| ChIPBax_reverse | GGGATCAGAGAGGAAC | BAX |
