# Supplementary figures and images for: A pro-apoptotic function of iASPP by stabilizing p300 and CBP through inhibition of BRMS1 E3 ubiquitin ligase activity
Source: Cell Death Dis. 2015 Feb 12;6(2):e1634–. doi: 10.1038/cddis.2015.17 (PMC4669821; doi:10.1038/cddis.2015.17)

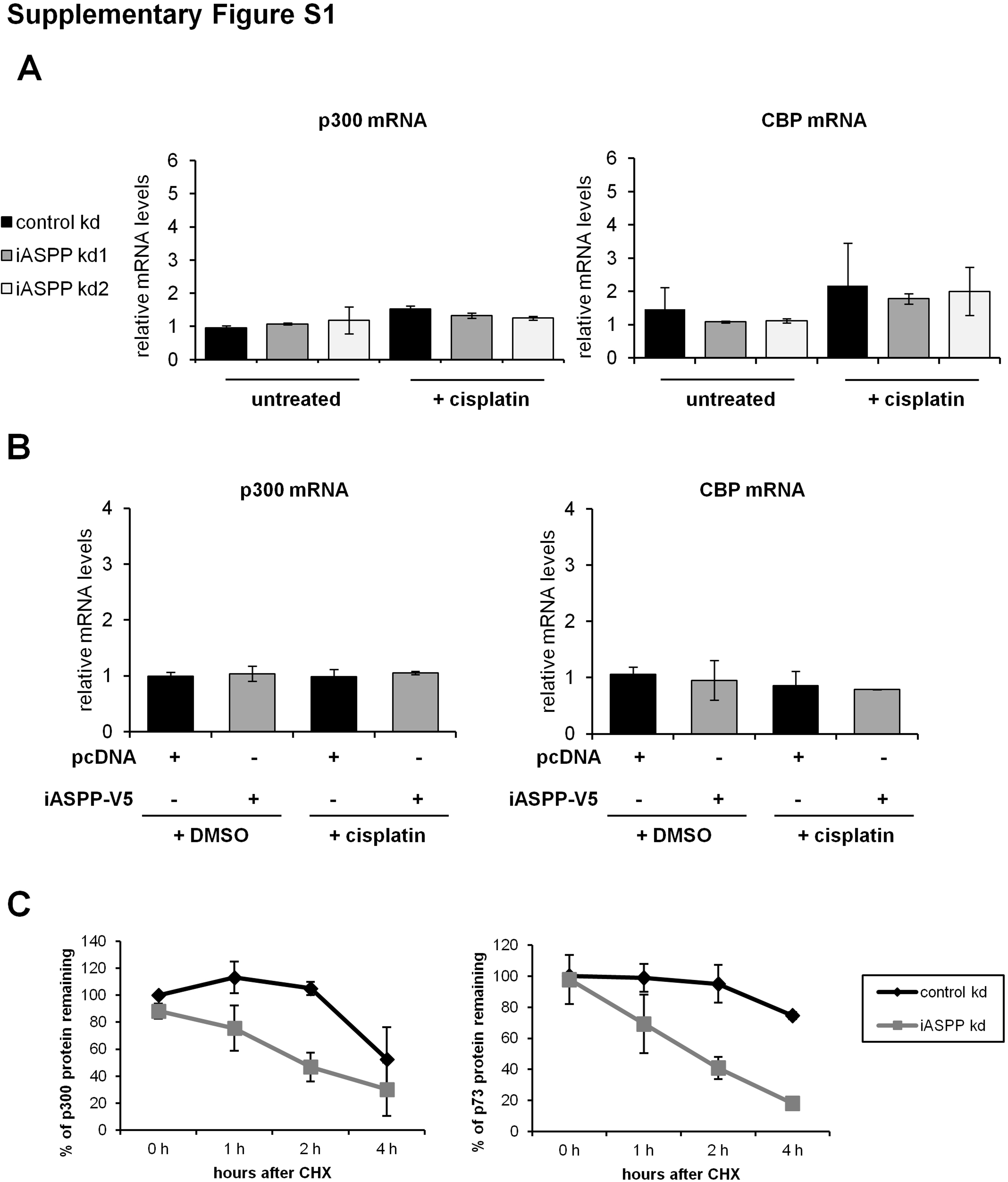

Supplement: Supplementary Figure 1 [file cddis201517x2.tif]

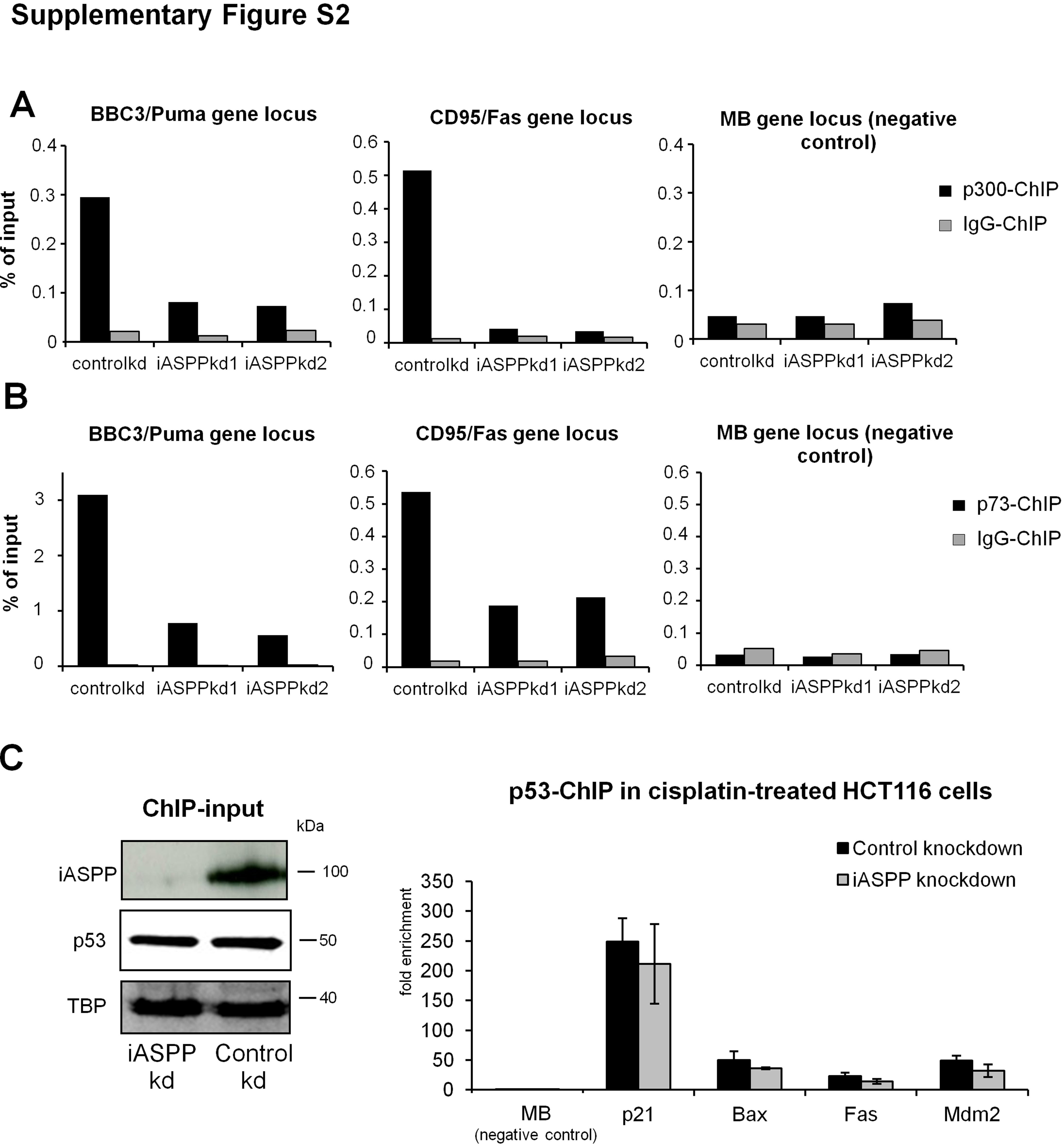

Supplement: Supplementary Figure 2 [file cddis201517x3.tif]

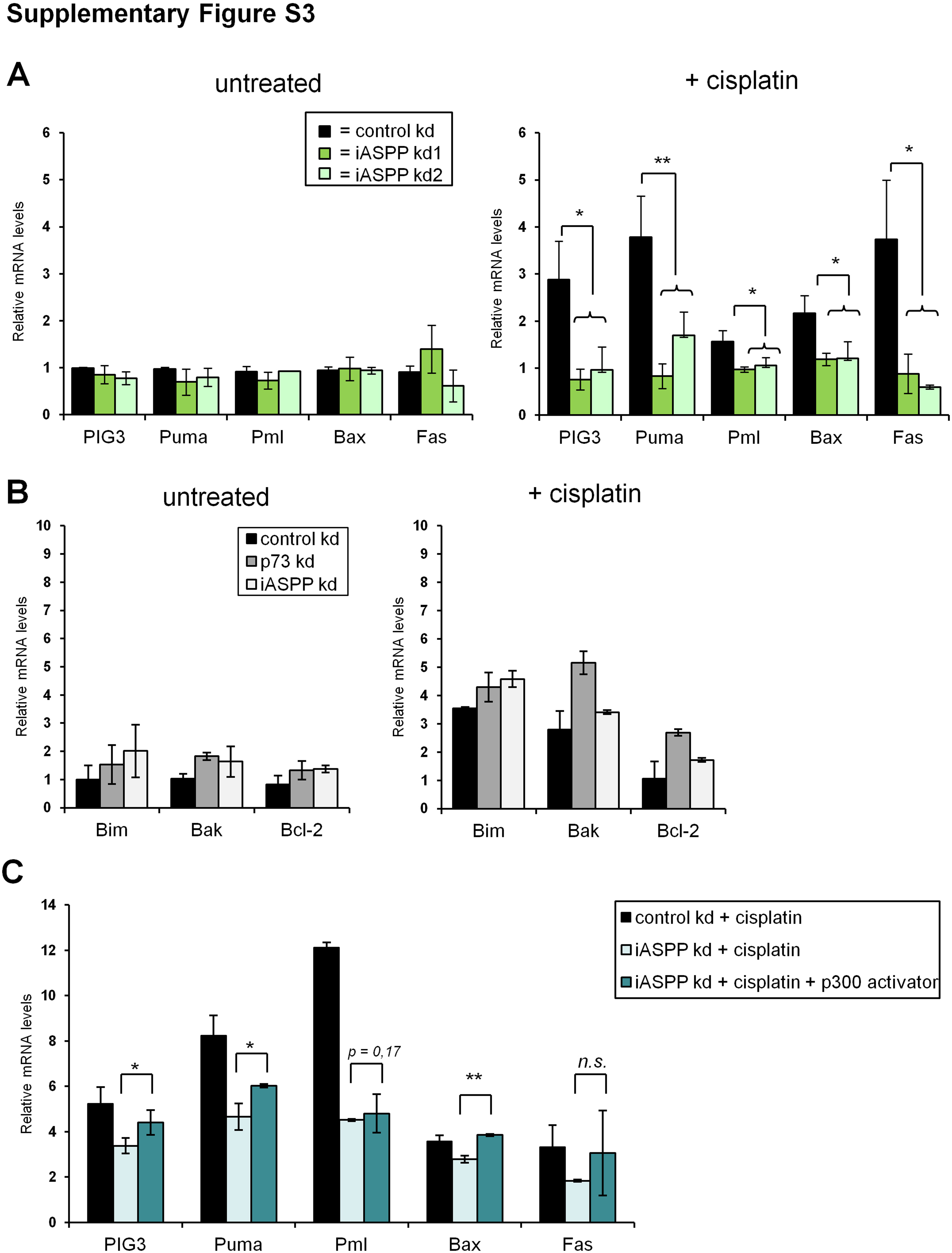

Supplement: Supplementary Figure 3 [file cddis201517x4.tif]

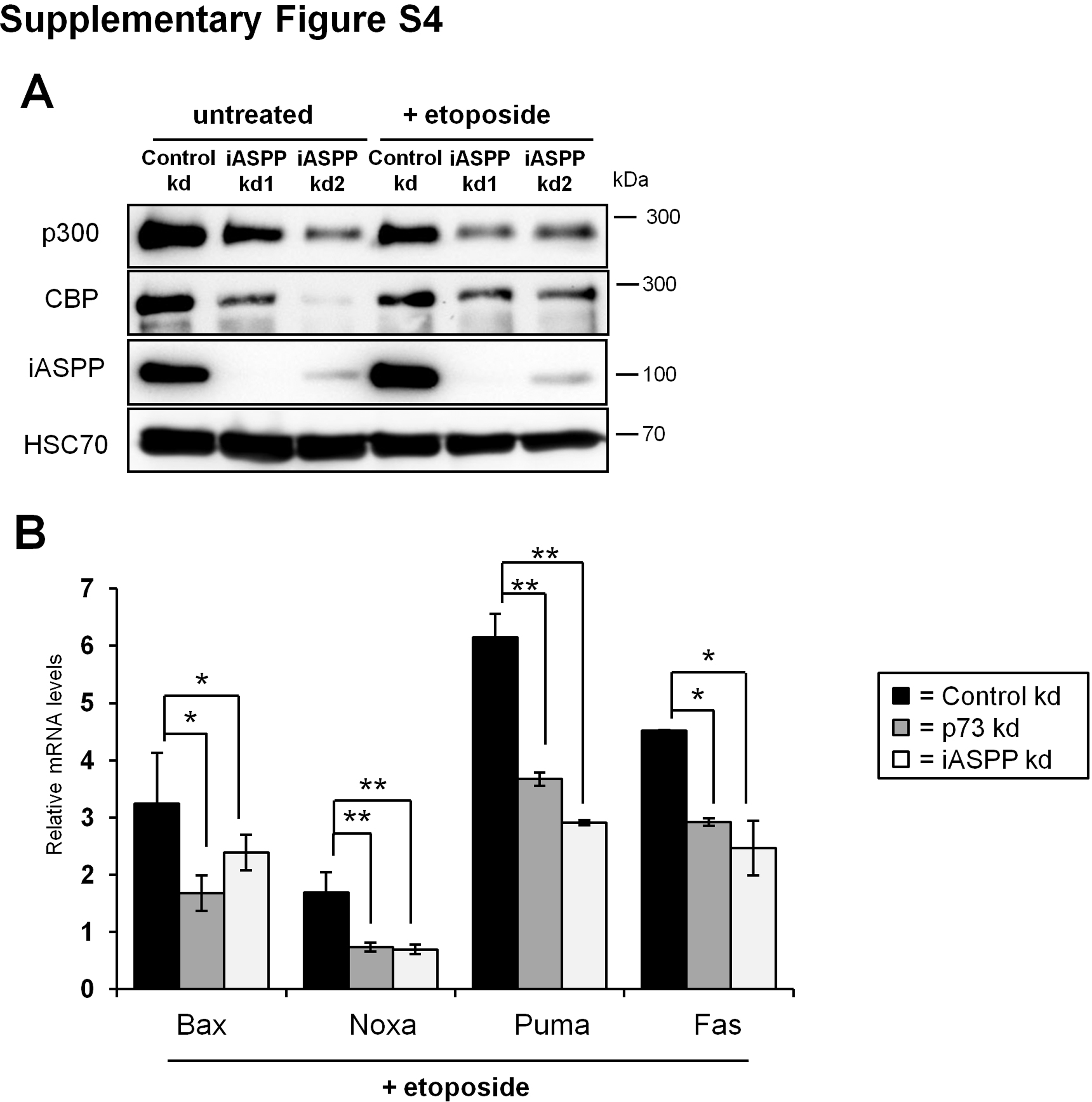

Supplement: Supplementary Figure 4 [file cddis201517x5.tif]

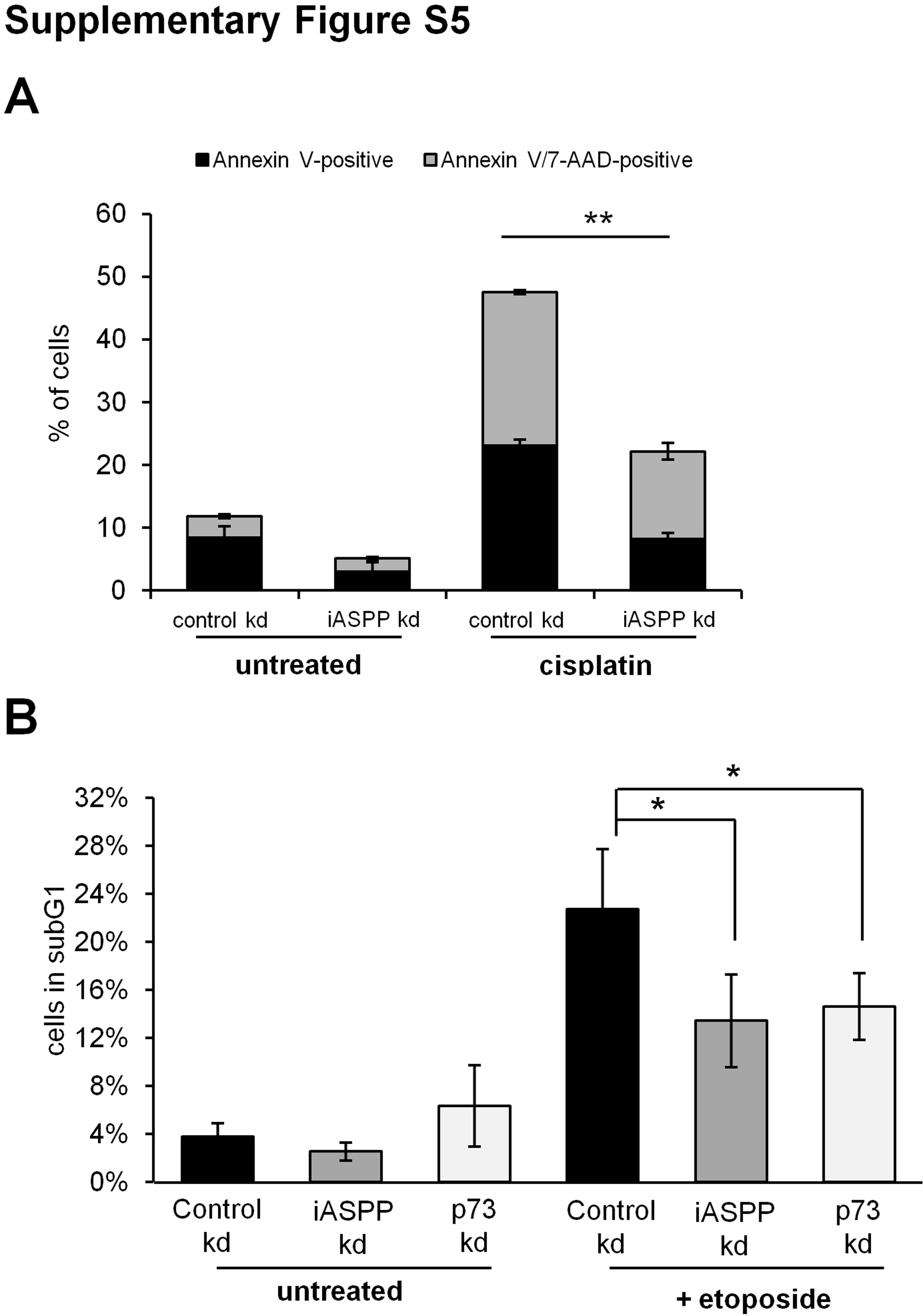

Supplement: Supplementary Figure 5 [file cddis201517x6.tif]

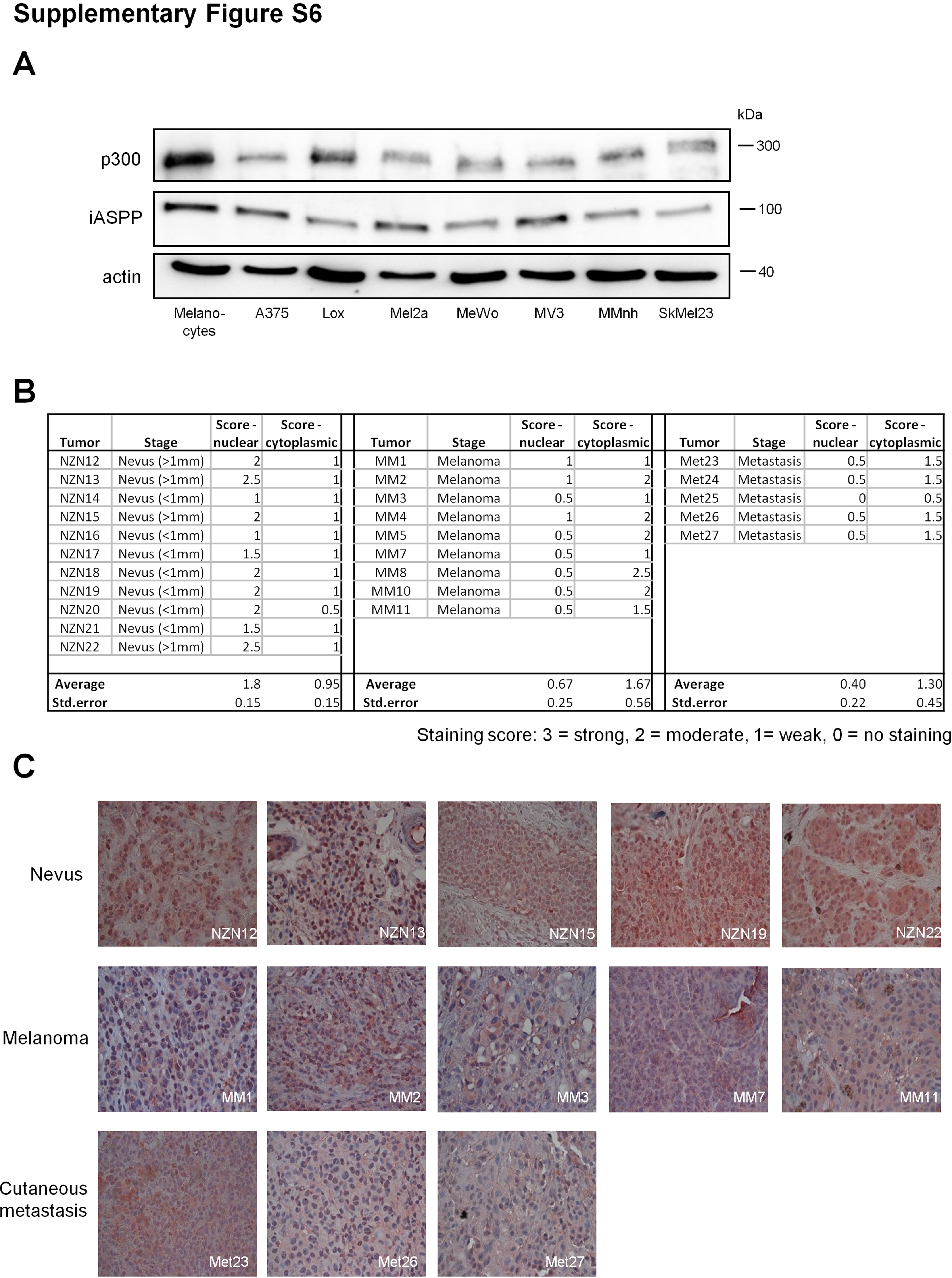

Supplement: Supplementary Figure 6 [file cddis201517x7.tif]

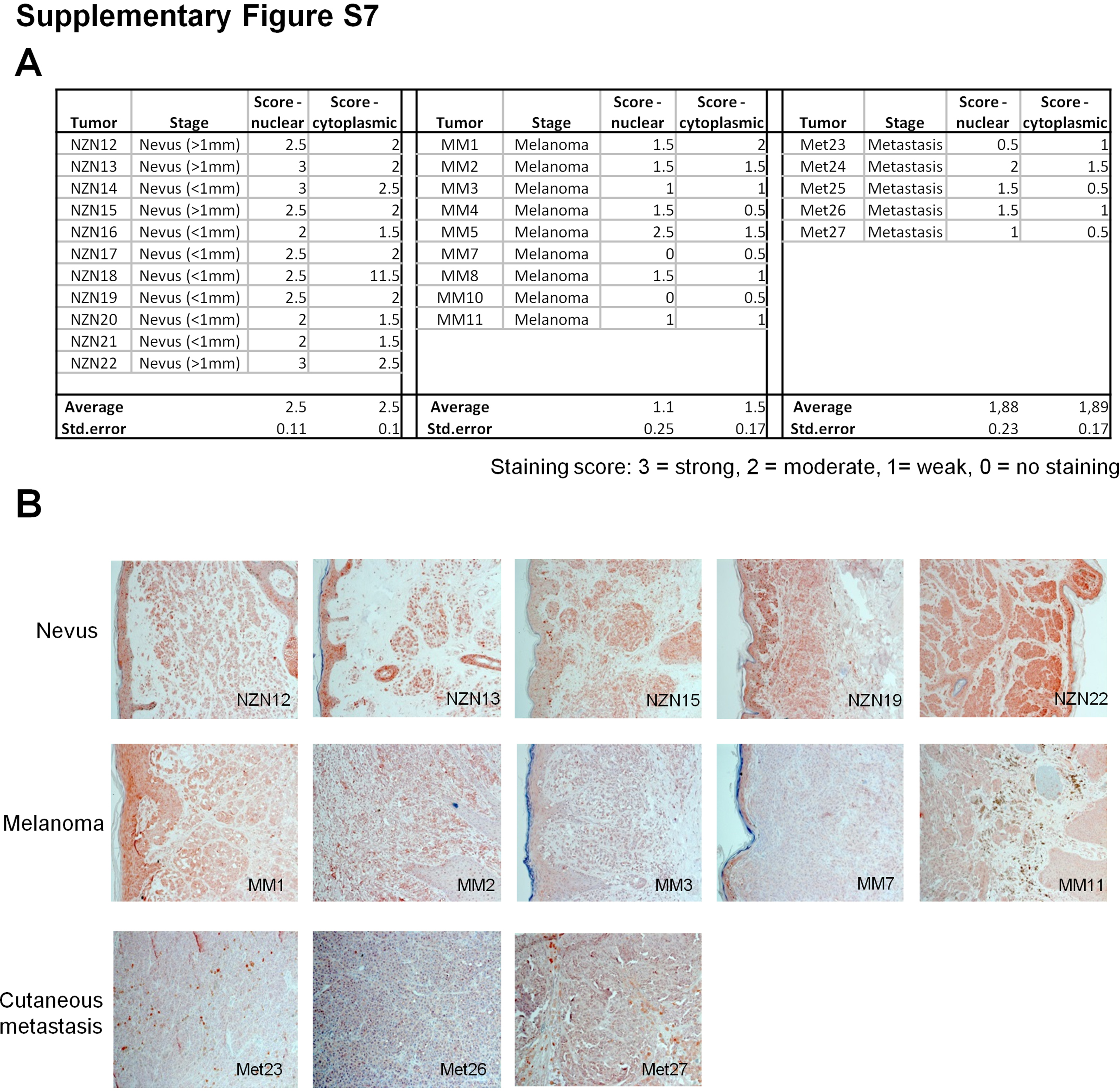

Supplement: Supplementary Figure 7 [file cddis201517x8.tif]

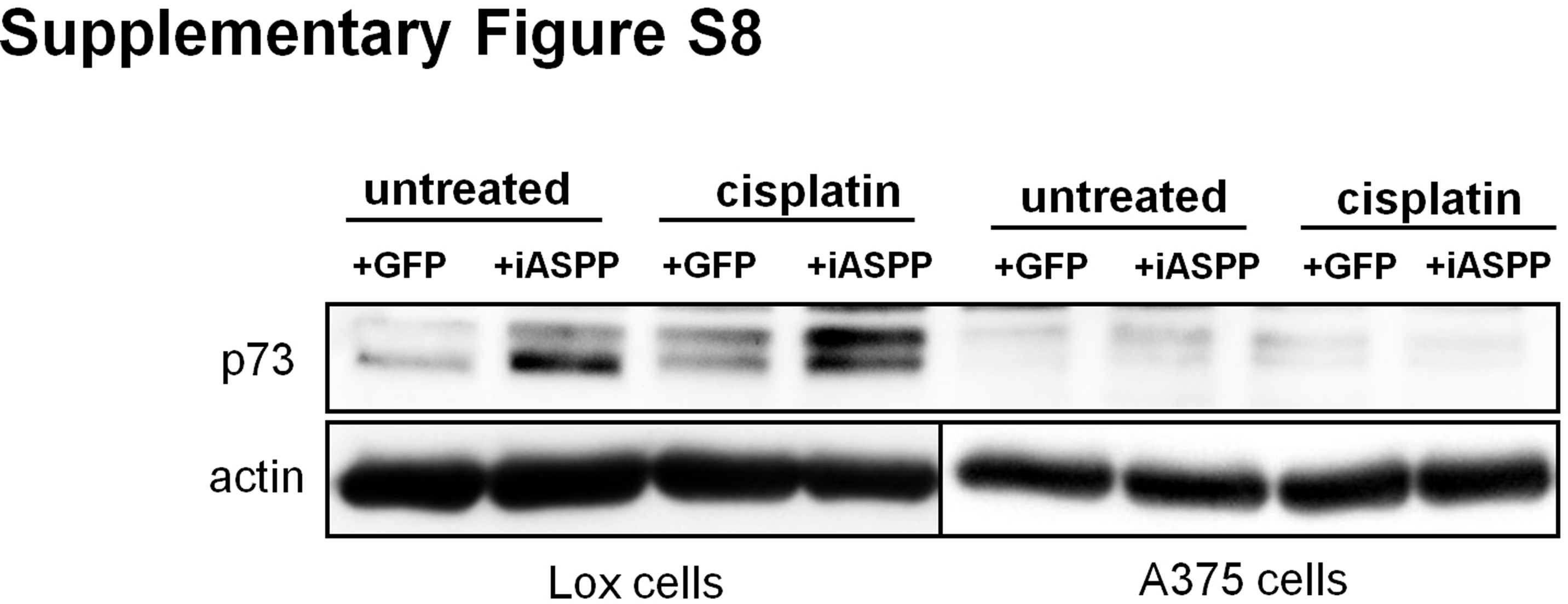

Supplement: Supplementary Figure 8 [file cddis201517x9.tif]
