# Supplementary material for: A pro-apoptotic function of iASPP by stabilizing p300 and CBP through inhibition of BRMS1 E3 ubiquitin ligase activity
Source: Cell Death Dis. 2015 Feb 12;6(2):e1634–. doi: 10.1038/cddis.2015.17 (PMC4669821; doi:10.1038/cddis.2015.17)
Supplement: Supplementary Figure Legends [file cddis201517x10.doc]

**Legends to supplementary figures**

**Supplementary Figure S1.** **Assessment of p300 and CBP mRNA levels, and quantification of p300 and TAp73 protein stability in iASPP-depleted HCT116 cells.**

**A.** Depletion of iASPP does not modulate the mRNA levels of p300 and CBP in untreated or cisplatin-treated HCT116 cells. Control and iASPP-depleted cells were treated with 20 µM cisplatin for 12 h, followed by RNA extraction. Gene expression was analyzed by cDNA synthesis and real-time qPCR, and values were normalized to HPRT1 expression. The bar graph represents the mean relative mRNA levels normalized to untreated control knockdown cells, along with the standard deviation from three independent biological replicas.

**B.** Overexpression of iASPP does not influence gene expression in DMSO- or cisplatin-treated cells. HCT116 cells were transiently transfected with a plasmid to express iASPP-V5 or an empty control vector (pcDNA3.1). 24 h after transfection, the cells were treated for 10 h with DMSO or 20 µM cisplatin. After RNA extraction and cDNA synthesis, gene expression was analyzed as in A. The bar graph represents the relative mRNA levels, normalized to DMSO-treated, pcDNA3.1-transfected cells.

**C.** Decreased stability of p300 and TAp73 in response to iASPP knockdown. HCT116 cells were treated for 20 h with 20 µM cisplatin, followed by addition of 100 µg/mL cycloheximide (CHX) for 0 h, 1 h, 2 h or 4 h. Immunoblots were analyzed by the Scion Image software (Scion coperation), and relative pixel values for each band corresponding to p300 or TAp73 were normalized to the corresponding pixel values of the loading controls. The relative percentages of the remaining p300 and TAp73 protein amounts are displayed along with the standard error (n=2), with the 0 h time points set to 100%.

**Supplementary Figure S2. Chromatin immunoprecipitation analysis of p300, TAp73 and p53 in cisplatin-treated HCT116 cells.**

**A.** Detailed representation of the p300-ChIP experiment. The bar graphs represent the percentage of input of the p300- and IgG-ChIP from the BBC3/Puma or CD95/Fas gene locus. Evaluation of the MB (myoglobin) gene locus served as a control. The data shown here derived from one experiment.

**B.** Detailed representation of the TAp73-ChIP experiment. Analysis and representation of the data are as in A.

**C**. Analysis of p53 DNA-binding characteristics in cisplatin-treated HCT116 cells. P53- or IgG-ChIP as control was performed in cisplatin-treated HCT116 cells (8 h, 20 µM cisplatin), followed by quantification of the percentage of precipitated DNA from specific genomic loci. The fold enrichment was calculated over a negative control (myoglobulin genomic locus, MB). Shown is the average binding from 3 independent experiments. For analysis of total p53 levels that localize to the chromatin fraction, input material for chromatin immunoprecipitation was subjected to immunoblot analysis.

**Supplementary Figure S3. Genes are coregulated by iASPP, p300 and TAp73, independent of p53.**

**A.** iASPP knockdown results in decreased pro-apoptotic target gene expression in p53 null cells. HCT116 -/-p53 cells, transduced to synthesize iASPP shRNA or control shRNA, were treated for 12 h with 20 µM cisplatin. Gene expression was analyzed by real-time qPCR. Values are normalized to the reference gene HPRT1. The bar graph represents the relative mRNA levels compared to untreated control knockdown cells. The p-value is calculated using the Student’s t-test and significance is indicated by asterisks as in Figure 2.

B. Bim, Bak and Bcl-2 mRNA levels are not downregulated by iASPP and p73 knockdown. In HCT116 cells containing control shRNA, p73 was transiently knocked down using siRNA. For control, scrambled siRNA was applied. 48 h after transduction, the cells were treated with 20 µM cisplatin for 16 h. In parallel, cells with a stable iASPP shRNA expression construct were treated and analyzed. After RNA extraction and cDNA synthesis, gene expression was analyzed by real-time qPCR. Values are normalized to the reference gene HPRT1. The bar graph represents the relative mRNA levels compared to untreated control knockdown cells.

C. Pretreatment of iASPP-depleted cells with the p300 activating drug CTB can partially re-establish the levels of pro-apoptotic target genes. Control or iASPP-depleted HCT116 cells were treated for 8 h with 100 µM CTB or DMSO, followed by addition of 40 µM cisplatin for 16 h. Subsequently, RNA was analyzed by real-time qPCR. Values were normalized to the reference gene HPRT1. The bar graph represents the relative mRNA levels compared to DMSO-treated control knockdown cells. The p-value was calculated using Student’s t-test, and the levels of significance are indicated by asterisks as in Figure 2.

**Supplementary Figure S4. iASPP enhances the activity of p300 and TAp73 in etoposide-treated cells.**

**A.** iASPP knockdown results in decreased p300/CBP protein levels upon etoposide treatment. After iASPP or control knockdown, HCT116 cells were treated with DMSO or 30 µM etoposide for 24 h, followed by immunoblot analysis.

**B.** Knockdown of iASPP or p73 results in a lower induction of pro-apoptotic p73 target genes in etoposide-treated cells. Upon stable transduction with control shRNA or shRNA against p73, HCT116 cells were treated with 30 µM etoposide or DMSO for 24 h. After RNA extraction and cDNA synthesis, gene expression was analyzed by real-time qPCR and normalized to the reference gene HPRT1. Significance was calculated using Student’s t-test and is illustrated by asterisks (* = p-value < 0.05, ** = p-value < 0.01, *** = p-value < 0.001).

**Supplementary Figure S5. iASPP modulates apoptosis in HCT116 -/- p53 cells and in etoposide-treated HCT116 cells.**

**A.** iASPP-depleted cells display lower levels of Annexin V staining upon cisplatin treatment. HCT116 -/-p53 cells were left untreated or incubated with 80 µM cisplatin for 24 h prior to harvest and staining of the cells with Annexin V/ 7-AAD solution. The percentage of unstained, Annexin V-positive and Annexin V/7-AAD-positive cells were determined using flow cytometry as in Figure S4.

**B.** iASPP contributes to p73-mediated apoptosis induction after etoposide treatment. Upon control, iASPP or p73 knockdown, HCT116 cells were treated with 30 µM etoposide or DMSO for 24 h. Subsequently, the cells were harvested, fixed with ethanol and stained with propidium iodide. The samples were subjected to flow cytometry, and the identical gate settings were applied for all samples. The bar graph depicts the percentage of cells with a DNA-content that corresponds to the sub-G1 phase. Asterisks indicate the significance of the differences as in Figure S4.

**Supplementary Figure S6. Assessment of p300, CBP and iASPP protein levels in melanocytes and quantification of CBP *in situ* staining in melanoma.**

**A.** p300 and iASPP protein levels are reduced in melanoma cell lines compared to primary melanocytes. Lysates from primary human epidermal melanocytes (NHEM, Promocell) and from seven untreated melanoma cell lines (A375, Lox, Mel2a, MeWo, MV3, MMnh and SkMel23) were immunoblotted and analyzed for protein levels of iASPP, p300 or actin as loading control.

**B.** Summary of the immunohistochemical analysis of melanoma tissue samples. The relative staining intensity score of nuclear and cytoplasmic CBP is displayed for all benign nevi, primary melanoma and cutaneous melanoma metastases samples. The score shows the relative intensity of the staining (3 = strong, 2 = moderated, 1 = low, 0 = no staining).

**C.** Images of selected immunohistochemical stainings of benign nevi, primary melanoma and cutaneous melanoma metastases. For staining, all samples were processed identically within the same experiments, and images were taken under standardized conditions (63x lens) and identical exposure times (Axiovision software).

**Supplementary Figure S7. Quantification of iASPP *in situ* staining in melanoma.**

**A.** Summary of the immunohistochemical analysis of melanoma tissue samples. The relative staining intensity score of nuclear and cytoplasmic iASPP is displayed for all benign nevi, primary melanoma and cutaneous melanoma metastases. The score shows the relative intensity of the staining (3 = strong, 2 = moderated, 1 = low, 0 = no staining).

**B.** Images of selected immunohistochemical stainings of benign nevi, primary melanoma and cutaneous melanoma metastases. For staining, all samples were processed identically within the same experiments, and images were taken under standardized conditions and identical exposure times (Axiovision software).

**Supplementary Figure S8. Increase of endogenous p73 levels upon restoring iASPP expression.**

P73 protein levels were detected from untreated and cisplatin-treated, control-transfected or iASPP-overexpressing A375 and Lox cells in parallel using same exposure times. For experimental details see Figure 7A.
